# Protocol for the three-dimensional analysis of rodent skeletal muscle

**DOI:** 10.1016/j.xpro.2024.103549

**Published:** 2025-01-10

**Authors:** Smrithi Karthikeyan, Yoko Asakura, Mayank Verma, Atsushi Asakura

**Affiliations:** 1 Stem Cell Institute, University of Minnesota Medical School, Minneapolis, MN, USA; 2 Paul & Sheila Wellstone Muscular Dystrophy Center, University of Minnesota Medical School, Minneapolis, MN, USA; 3 Department of Neurology, University of Minnesota Medical School, Minneapolis, MN, USA; 4 Department of Pediatrics & Neurology, Division of Pediatric Neurology, The University of Texas Southwestern Medical Center, Dallas, TX, USA

**Keywords:** classification Description: cell biology, developmental biology, model organisms, molecular biology, stem cells, cell differentiation

## Abstract

Confocal imaging is a powerful tool capable of analyzing cellular spatial data within a given tissue. Here, we present a protocol for preparing optically cleared extensor digitorum longus (EDL) skeletal muscle samples suitable for confocal imaging/computational analysis. We describe steps for sample preparation (including perfusion fixation and tissue clearing of muscle samples), image acquisition, and computational analysis, with sample segmentation/3D rendering outlined. This protocol can be applied to characterize various cell types, including muscle satellite cells (muscle stem cells) and capillary endothelial cells within rodent skeletal muscle.

For complete details on the use and execution of this protocol, please refer to Verma et al.,^1^ Verma et al.,^2^ Karthikeyan et al.,^3^ and Karthikeyan et al.^4^

## Highlights

• 3D imaging analysis for muscle stem cells and capillaries in mouse skeletal muscle
• Tissue clearing techniques for mouse skeletal muscle samples
• 3D skeletal muscle image acquisition by confocal microscope
• Data analysis by using Fiji/Ilastik and Imaris software for 3D image analysis

## Abstract (editor highlights)

Publisher’s note: Undertaking any experimental protocol requires adherence to local institutional guidelines for laboratory safety and ethics.

## Graphical abstract

**Figure:**
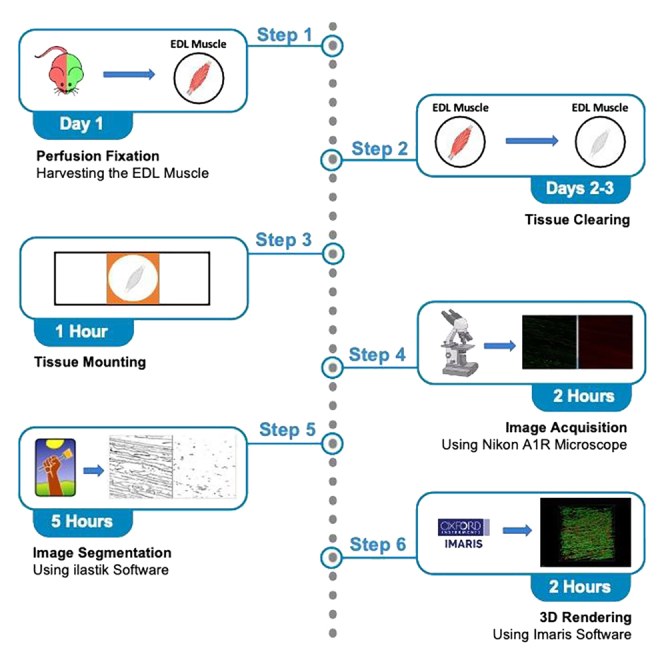

## Before you begin

Skeletal muscle is responsible for important functions, such as voluntary movement, postural maintenance, and respiration. The important characteristics of skeletal muscle are its remarkable regenerative capacity, adapting to physiological needs, including growing mass, training, and damage.^5^^,^^6^ Skeletal muscle contains a variety of cell types, including muscle fibers, satellite cells (MuSCs)/skeletal muscle stem cell population, peripheral nerves, Schwann cells, immune cells, pericytes, endothelial cells (ECs), and fibrillar progenitor cells (FAPs).^7^^,^^8^^,^^9^ The cellular organization and cell-cell interactions exist in the complex cellular network are altered from their basal state during developmental and regenerating processes.^1^^,^^2^^,^^3^^,^^4^ A better understanding of the structure, function, and interactions of the cells within the network is essential to understand how the cells within the network interact with one another during the regenerative process. Better understanding of cell-cell interactions within muscle, the muscle structure, and the muscle function will direct to a more comprehensive understanding of the process of the muscle regeneration. Therefore, to accurately understand the vital cellular interactions within muscle, the muscle tissues must be characterized in three dimensions (3-D).

3-D analysis by light cleavage using confocal microscopy is a promising strategy. Traditionally, there are several limitations for these approaches, including the existence of light-scattering molecules, including endogenous fluorescent molecules lipids (NADH and myoglobin), extracellular matrix and lipids.^1^ However, several protocols for optical tissue clearing, such as CLARITY,^10^ CUBIC,^11^ DISCO,^12^ and other strategies, have attempted to overcome the existing problems. The definitive protocol for skeletal muscle tissue clearing is to reduce background signals from dyes and fluorescence molecules, refractive index mismatch-induced light scattering, and is to be compatible to immunostaining.^1^ In this protocol, we illustrate the confocal imaging strategy for skeletal muscle tissue to achieve the above goals. We propose an upgraded optical skeletal muscle tissue cleanup protocol for sample preparation. Advances in imaging and computer technology have made the analysis of spatial data from confocal microscopy images increasingly powerful. This protocol is intended to perform confocal microscopic imaging for computational 3-D rendering. The overall process involves segmentation and 3-D rendering of the captured microscopic images using Ilastik and Imaris software.^13^^,^^14^

The following protocol describes specific procedures for optical tissue clearing, confocal image acquisition, and 3-D rendering of skeletal muscle cross-sectional images of adult mice in order to produce image models of skeletal muscle sections suitable for data acquisition.

### Institutional permissions

University of Minnesota Research Animal Resources (RAR) monitors animals housed by SPF environment. The Institutional Animal Care and Usage Committee (IACUC) approved protocols used for this project at the University of Minnesota. NIH guidelines were used for the animals in this project.

## Key resources table

**Table:** 

REAGENT or RESOURCE	SOURCE	IDENTIFIER
**Chemicals, peptides, and recombinant proteins**		

Acrylamide	MilliporeSigma	A9099
Boric acid	MilliporeSigma	B6768
Corn oil	MilliporeSigma	C8267
Diatrizoic acid	MilliporeSigma	D9268
Ethyl alcohol	MilliporeSigma	EX0280
Heparin	MilliporeSigma	H3149
Iodixanol/Histodenz/Omnipaque	MilliporeSigma	PHR3015
Isoflurane	VWR	AAAL17315-14
n-Methyl-d-glucamine	MilliporeSigma	M2004
N, N, N′, N′-TETRA-KIS (2-hydroxypropyl) ethylenediamine	MilliporeSigma	122262
Paraformaldehyde (PFA)	MilliporeSigma	P6148
Phosphate-buffered saline (PBS)	MilliporeSigma	P2272
Sodium Azide (NaN_3_)	MilliporeSigma	S2002
Sodium dodecyl sulfate (SDS)	MilliporeSigma	74255
Sodium hydroxide (NaOH)	MilliporeSigma	S5881
Tamoxifen (TMX)	MilliporeSigma	T5648
Tween-20	MilliporeSigma	P1379
Urea	MilliporeSigma	U5378
VA-044	FUJIFILM Wako Chemicals	NC0632395

**Experimental models: Organisms/strains**		

*Flk1*^*GFP*^ mice (*Kdr*^*tm2.1Jrt*^*/J*), 2 months old (male or female)	Jackson Laboratory	#017006
*Pax7*^*CreERT2*^ mice (*B6.Cg-Pax7*^*tm1 (cre/ERT2) Gaka/*^*J*), 2 month-old (male or female)	Jackson Laboratory	#017763
*R26R* mice (*B6.Cg-Gt (ROSA)26Sortm9 (CAG-tdTomato) Hze/J*), 2 months old (male or female)	Jackson Laboratory	#007909

**Software and algorithms**		

Fiji ImageJ (NIH): For segmentation, the HDF5 and Ilastik plugins must be installed	NIH	https://imagej.net/software/fiji/downloads
Ilastik (1.4.0): For segmentation, any image files must be converted into an HDF5 file in order for the software to read the file properly	Ilastik.org	https://www.ilastik.org/download.html
Imaris	Oxford Instruments	https://imaris.oxinst.com/packages
Software (NIS Elements)	Nikon	https://www.nikoninstruments.com/Products/Software/NIS-Elements-Advanced-Research

**Other**		

KDS single-syringe pump, series 100	MilliporeSigma	Z401358
Nikon A1R FLIM and FCS confocal microscope	Nikon	N/A
25-gauge needle	MilliporeSigma	Z192406
15 mL Conical tube	Fisher Scientific	05-527-90
Cover glass	MilliporeSigma	CLS2975245
Microdissection scissors	Stevens Tenotomy	No. 14064-11
Parafilm	MilliporeSigma	P7668
Silicone isolator (1.6 mm depth)	MilliporeSigma	GBL664204
Silicone isolator (2.4 mm depth)	MilliporeSigma	GBL664304
Syringe (1 mL)	MilliporeSigma	Z683531
Syringe (50 mL)	MilliporeSigma	XX1105005
Tweezer (Dumont #5)	Fisher Scientific	50-822-208
Tubing	Fisher Scientific	50-199-5222

## Materials and equipment

**Table:** Fixation buffer (2% para-formaldehyde [PFA] buffer)

Reagent	Working solution	Weight or volume
Para-formaldehyde (PFA)	2%	4 g
3M NaOH	–	few drops
20 × PBS	1 × PBS	10 mL
ddH_2_O	–	190 mL
**Total**	**–**	**200 mL**

3M NaOH (3M) is added to dissolve the powder at 60°C. 20 × PBS (5 mL) is added after dissolving the powder to adjust the pH to 6.9. The working solution is cooled down to 4°C (use within 1 month).

**Table:** A4P0 solution

Reagent	Working solution	Weight or volume
Acrylamide	4%	4 g
VA-044	0.25%	0.25 g
PBS	1 × PBS	100 mL
**Total**	**–**	**100 mL**

The solution should be kept at −20°C (use within 10 months).

Prepare the solution on ice. Aliquot in 15 mL tubes. The solution should be stored at −20°C. This reagent contains acrylamide which is a potent neurotoxin. Wear personal protection equipment, such as lab coats, goggles and gloves.

**Table:** PBSTT

Reagent	Working solution	Weight or volume
PBS	1 × PBS	499 mL
Triton X-100	0.1%	0.5 mL
Tween-20	0.1%	0.5 mL
NaN_3_ (sodium azide)	0.01%	0.05 g
**Total**	**–**	**500 mL**

The solution should be kept at 4°C (use within 10 months).

**Table:** Clearing Solution 1 (CS1)

Reagent	Working solution	Weight or volume
Boric acid	20 mM	100 mL
SDS	5%	5 g
3M NaOH	–	few drops
**Total**	**–**	**100 mL**

3M NaOH is used to adjust the pH to 9.4. The solution should be kept at 4°C (use within 10 months).

**Table:** Clearing Solution 2 (CS2)

Reagent	Working solution	Weight or volume
Boric acid	20 mM	90 mL
N,N,N′,N′-tetra-kis(2-Hydroxypropyl)ethylenediamine	10%	10 g
Urea	10%	10 g
Triton X-100	10%	10 mL
HCl	–	few drops
**Total**	**–**	**100 mL**

For reduction of the viscosity, the reagents should be incubated in a 60°C for 25 min. Following removal of the SDS, skeletal muscle tissue is kept at 4°C. HCl is used to adjust the pH adjusts to 9.4. The solution should be kept at 4°C (use within 10 months).

**Table:** PROTOS

Reagent	Working solution	Weight or volume
n-methyl-d-glucamine	23.5% (w/v)	47 g
Diatrizoic acid	29.5% (w/v)	59 g
Iodixanol (Omnipaque or Histodenz)	32.5% (w/v)	65 g
NaN_3_ (sodium azide)	0.01%	0.02 g
ddH_2_O	–	adjust to 200 mL
**Total**	**–**	**200 mL**

To prevent liquid leak, Parafilm should be used for the tube cap for PROTOS solution due to its very volatile feature. The solution should be kept at 4°C (use within 1 year). Adapted from Asakura et al.^9^

20 mg/mL Tamoxifen (TMX) solution: add 1 g Tamoxifen in 50 mL Corn Oil and shaking in 37°C shaker overnight. The solution should be stored at −80°C.

## Step-by-step method details

### Tamoxifen injection-day −5

**Timing: 10 min**

Before Perfusion Fixation, *Pax7^tdTomato^* (*Pax7*^*CreERT2*^*:R26R*)*:Flk1^GFP^* mice were injected with TMX every day for 3–5 times. The overall workflow of this protocol is schematically shown in the graphical abstract.

***Note:*** 50 mL corn oil is used for dissolving 1 g of TMX in the shaker incubator (37°C) overnight, and the TMX solution is kept in −80°C. Adult mice (2–4 months old) are intraperitoneally injected with 200 μL of TMX (20 mg/mL) / 20 g body weight.

### Perfusion fixation and dissection-day 1

**Timing: 4–6 h**

This section below details a perfusion fixation/dissection protocol for harvesting the EDL muscle from mice.

**Figure 1:**
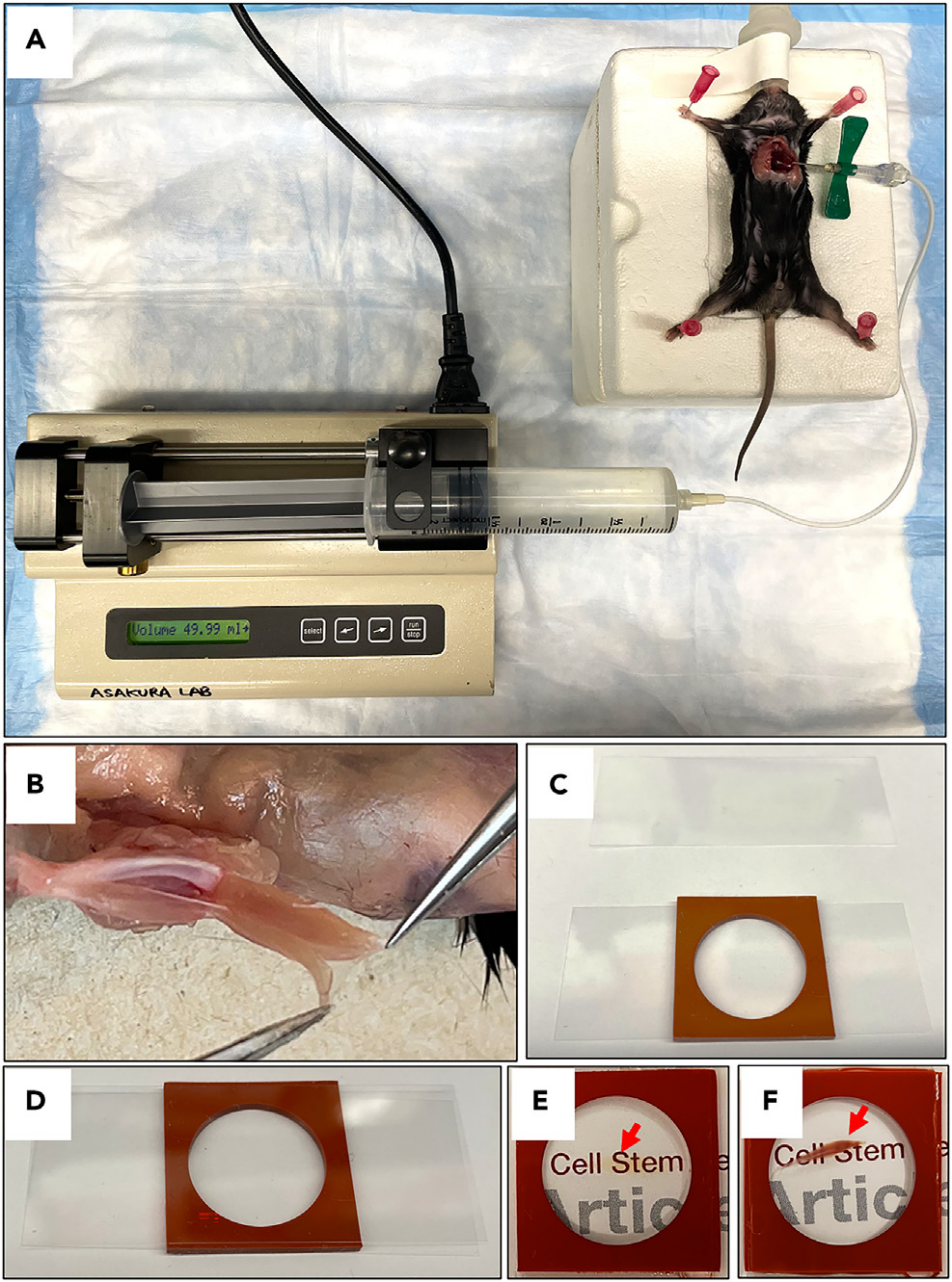
Perfusion fixation, tissue dissection and mounting EDL muscle (A) Following anesthetization, TMX-treated *Pax7*^*tdTomato*^*:Flk1*^*GFP*^ mice were perfusion-fixed with a syringe pump. (B) Perfusion-fixed mice had their hindlimb skin excised and the EDL muscle detached from the hindlimb muscle. (C and D) Silicon isolator with cover glasses was used for tissue-mounting. (E and F) The tissue clearing process made the EDL muscle transparent (E), compared with the EDL muscle without tissue clearing (F).

1. **Day 1:** Sedate the mouse using isoflurane, the most used inhalational anesthetic compound. A fixation chamber is used for pined mice. A cone is placed over the nose of the pined mouse to provide constant isoflurane during opening of the chest cavity to ensure the animal remains sedated.
2. To expose the heart, the chest cavity is opened. Use pins to hold the ribs in place. A needle is inserted into the left ventricle of the heart and fixed. Connect the needle to the tube for a syringe pump (Figure 1A).
3. Perfuse with 5 mL PBS with 2 mL/min flow speed followed by 50 mL Fixation Buffer with 2 mL/min flow speed.

**CRITICAL:** If the mouse's tail is seen moving, perfusion is considered to be successful. It is critical that in order for PBS to flow properly into the muscle, the heartbeat must be observed at the beginning of this process.

4. The extensor digitorum longus (EDL) muscle is detached as follows (Figure 1B).
  a. Carefully lift the EDL tendon end next to the tibialis anterior (TA) tendon with forceps.
  b. Cut as close to the foot as possible.
  c. Release the EDL muscle from the other muscles until the upper tendon is visible.
  d. Sever the upper tendon.
5. Postfix the tissue at 4°C with overnight rocking.

### Tissue clearing-day 2

**Timing: 2 h**

The section below outlines a protocol for optically clearing the harvested EDL muscle tissue to be suitable for confocal imaging.

6. Wash three times with ice-cold PBS containing 0.02% sodium azide (NaN_3_).
7. Thaw the A4P0 solution on ice. Ensure that the temperature of the solution does not rise significantly.

***Note:*** A4P0 solution: Dissolve 4% acrylamide and 0.25% VA-044 in 50 mL PBS: Dissolve 2 g acrylamide and 0.125 g VA-044 in 50 mL PBS on ice. Store the solution at −20°C.

8. Incubate tissues overnight at 4°C in cold A4P0 with shaking.

### Tissue clearing-day 3

**Timing: 2 h**

The section below outlines a protocol for optically clearing the harvested EDL muscle tissue to be suitable for confocal imaging.

9. Degas A4P0 solution on ice.
  a. Thaw 16 mL of A4P0 and transfer to a 50 mL plastic tube.
  b. Bubbling nitrogen gas into the liquid for 3 min.
  c. Allow the solution to precipitate until there are no more bubbles in the solution. This should occur in about 1 min.
10. Remove A4P0 solution with tissue and collect waste for proper disposal.
11. Transfer the degassed A4P0 to a 5–15 mL tube containing the sample. Overfill and allow a meniscus to form on top of the tube. Ensure that no air bubbles form in the tube.
  a. Lightly cover the tubing with 1"×1" plastic wrap to ensure there are no air bubbles.
  b. Screw the cap tightly onto the top of the tubing to allow excess liquid to drip into the waste stream.
12. Incubate at 37°C using a tube rotator to polymerize acrylamide. Polymerize in light-shielded tubes (aluminum foil) for 3 h.
13. Remove the tissue from the A4P0 solution and discard properly.
14. Wash three times at 37°C for 30 min each in PBSTT.

***Note:*** PBSTT: 500 mL PBS with 0.1% Triton X-100, 0.1% Tween-20, and 0.02% NaN_3_ (sodium azide).

15. Transfer samples to the Clearing Solution 1 (CS1) overnight at 37°C with rocking.

***Note:*** Clearing Solution 1 (CS1): 5% SDS in 20 mM boric acid buffer. pH of the solution is about 8.0 after dissolution. Adjust pH to 9.0–9.5 by adding a few drops of 3M NaOH.

**Pause point:** EDL muscles are left at 4°C.

### Tissue clearing-day 4

**Timing: 1 h**

The section below outlines a protocol for optically clearing the harvested EDL muscle tissue to be suitable for confocal imaging.

16. Wash three times with PBSTT with rocking for 30 min each at 37°C.
17. Incubate the tissue in Clearing Solution 2 (CS2) overnight at 37°C with rocking. After overnight incubation, the tissue should appear clear now.

***Note:*** Clearing Solution 2 (CS2): 10% N,N,N′,N′-tetra-kis(2-hydroxypropyl)ethylenediamine, 10% urea, 10% Triton X-100 in 20 mM boric acid: warm all reagents in an incubator at 50°C–60°C for 30 min to reduce viscosity. This removes SDS and keeps the muscle tissue at 4°C. After lysis, the solution should have a pH of about 10.8. Adjust the pH to 9.0–9.5 by adding a few drops of hydrochloric acid.

### Tissue clearing-day 5

**Timing: 4 h**

The section below outlines a protocol for optically clearing the harvested EDL muscle tissue to be suitable for confocal imaging.

18. Wash three times with PBSTT at 37°C, rocking for 30 min each.
19. Incubate the EDL muscle in PROTOS^9^ three times for 30 min each at 37°C with rocking.
20. Perform multi-view imaging as described below using the PROTOS solution and embedding method.
21. Press approximately 1 inch of tape against one side of the silicon isolator to remove debris (Figures 1C and 1D).
22. Press the spacer firmly onto the center of the cover glass and affix the clean side of the isolator (Figure 1D).
23. Press another piece of tape against the exposed side of the isolator to remove any remaining dust (Figure 1D).
24. Place the sample (in PROTOS solution) into the isolator using tweezers (Figures 1E and 1F).
25. Inject the remaining PROTOS solution with a pipette until the muscle sample is immersed in the isolator (Figures 1E and 1F).
26. Attach the top cover glass by pressing it onto the spacer/glass complex below (Figures 1E and 1F).

***Note:*** Make sure there are no bubbles in the isolator.

### Image acquisition-day 5

**Timing: 2 h**

This section describes how to image the tissue cleared muscle sections using the Nikon A1R microscope. In general, a confocal microscope is required to image cleared tissue samples. To select the ideal confocal microscope lens, the working distance of the lens and the thickness/size of the muscle sample must be known. For EDL tissue-cleared muscles, the 20× water immersion lens on the Nikon A1R provides precise results. Usually, water immersion lenses provide more transparent images for two-photon confocal imaging. The images of the skeletal muscles’ vasculature and stem cells were taken using GFP and mCherry filters (mCherry has a similar excitation/emission maxim to tdTomato).

**Figure 2:**
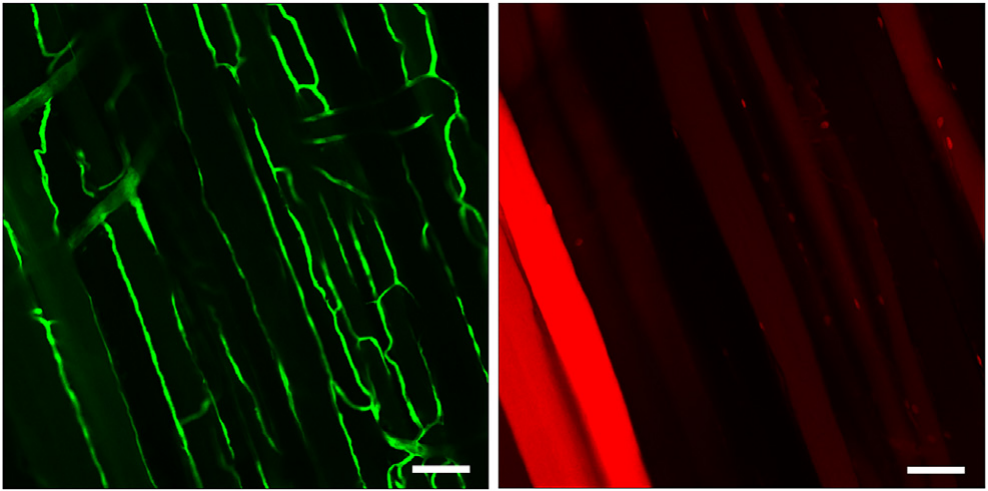
Confocal images without post-processing of EDL muscle Successful confocal microscopy of EDL muscle. GFP(+) vascular ECs (left) and tdTomato(+) MuSCs (right). The right panel shows some of the tdTomato(+) muscle fibers generated by the fusion of tdTomato(+) MuSCs with existing muscle fibers. Scale bars; 50 μm.

27. Switch on the 488 nm (GFP) and 561 nm (mCherry) lasers with the laser controller.
28. Set the pinhole diameter of the shortest wavelength laser (488 nm laser) to 1.2 μm. This can be adjusted in the A1 Compact GUI window.
29. In the A1 Compact GUI window, select an image size of 1024 × 1024 pixels.
30. In the Scanning Window, select a Z range of 400 μm (401 steps).
31. Turn on the confocal microscope according to the facility’s instructions. Ensure that the 488 nm and 561 nm lasers are turned on with the laser controller.
32. Select a 20× water immersion objective on the microscope. Drip 2–3 drops of distilled water onto the lens using a syringe.
33. Place the mounted specimen slide on the stage.
34. Select the "Eyepiece-EPI" tab in NIS Elements Software. Use the microscope eyepiece to locate the sample. Select mCherry or GFP in the menu and turn on the corresponding wavelength laser.
35. Use the microscope to locate a region of the sample that is suitable for imaging (MuSCs are visible with mCherry and vascular ECs are visible with GFP).
36. Move the laser intensity tabs for each laser (mCherry and GFP) in the A1 Compact GUI window and adjust the image brightness for both channels. Adjust so that the spots in the image are barely saturated.
37. Because the sample is three-dimensional, the user must identify the top and bottom of the sample in order for the microscope to capture an image. Adjust Z with the side knob on the microscope until the top of the sample is reached.
38. Once the top of the sample is reached, press "Top" in the scan window. Manually enter the corresponding bottom surface so that the total depth of the image is 400 μm.
39. In the A1 Compact GUI window, select the desired image size for the 1024 pixel scan area.
40. Press "Run Now" in the scan window to capture the image. A new window will pop up and display the image acquisition time.
41. After taking the image, press "File" > "Save" As to save the image in the ND2 file format to the desired location.
42. To convert the image file to TIF/TIFF format for analysis, open the ND2 file in FIJI ImageJ (9, 10). Once open, select "File" > "Save As" > "Tiff". Confocal Images without post-processing of EDL muscle was shown in Figure 2.
43. Store samples in PROTOS after imaging at 4°C.

### Image segmentation-day 6

**Timing: 5 h**

The overall process of creating a 3D rendering of a microscopic image involves segmenting the original microscopic image using the software ilastik and creating a 3D rendering using Imaris^2^^,^^4^ Segmentation is an image processing process that transforms an image into a binary image. Several different segmentations software have been developed, but this protocol uses ilastik because of its ease of use and powerful segmentation algorithm.^13^^,^^14^ After segmentation is complete, the segmented binary image can then be 3-D rendered/analyzed through the visualization software Imaris. The below protocol describes how to segment the acquired images into binary images using Fiji ImageJ ilastik.

44. General Protocol for Image Segmentation Using FIJI ImageJ and Ilastik.
  a. Import the raw image file (.nd2 file type) into FIJI ImageJ.
  b. Convert and save the image as a TIF/TIFF file type: “File” > “Save As” > “Tiff…”
45. For multichannel images, split the image channels of the TIF file: “Image” > “Color” > “Split Channels”.
46. Use the HDF5 plug-in to save each channel in HDF5 format: “File” > “Save As” > “HDF5” > “Save” to HDF5 (new or replace).
47. To reduce the computational load of the ilastik software, it is useful to crop large image files into smaller images; in the case of the 1024 × 1024 × 401 image size (used in this protocol), part of the image was cropped before loading it into ilastik.

***Note:*** Crop procedure in Fiji: “Image” > “Crop” using the Rectangle Crop tool on the toolbar. Crop at least 5 different 50 × 50 × 401-pixel images from each channel.

48. Save each cropped image in HDF5 file format (same protocol as 42).
49. Start a new pixel classification project file in Ilastik.
50. Open all cropped image files (all in .h5 file format) in the Input Data tab: Add “New” > Add “Separate” Image.
51. In the Feature Selection tab, select all available features: Select “Features” > “Drag” with the mouse and check all boxes.
52. In the Training tab, create two labels. One label will be designated as the background and the other will be defined as the feature of interest. In this protocol, the feature of interest was either the MuSCs or the blood vessel ECs.
53. After selecting a starting label (either background or feature of interest), select the paint brush tool.
  a. Identify parts of the image that are either the background or the feature of interest.
  b. After identifying some spots, press Live Update with only the Segmentation box checked.

***Note:*** This will now show a segmented version of the cropped image. This step should be repeated until the segmentation is as accurate as possible.

**Figure 3:**
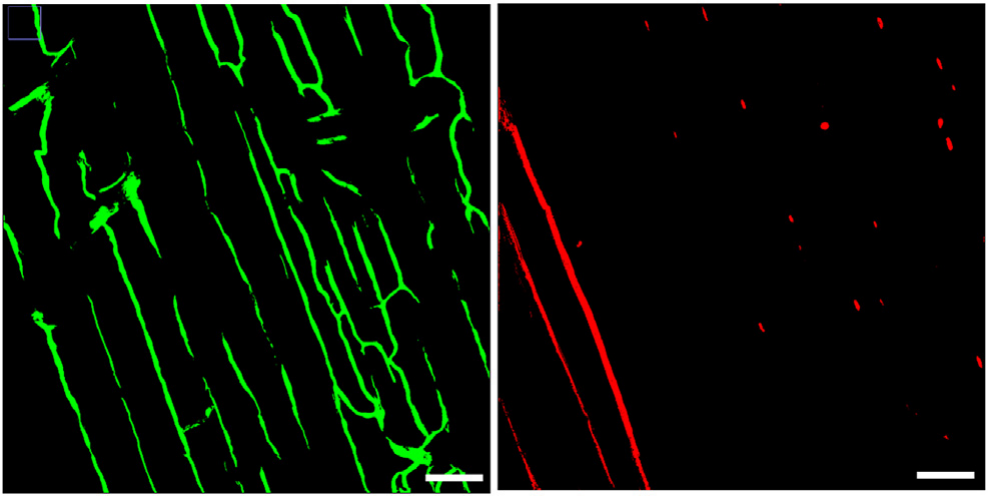
Image segmentation results using Ilastik Results of Figure 2: successful image segmentation of EDL muscle. GFP(+) vascular ECs (left) and tdTomato(+) MuSCs (right). Scale bars; 50 μm.

54. Repeat step 53 for each cropped image.
55. In the Prediction Export tab, change the Source to “Simple Segmentation”
56. In the Batch Processing tab, select the complete .h5 file of one of the channels of the original image by clicking the “Select Raw Data Files” button. Then, to segment the entire channel, click “Process all Files”.
57. Once the segmentation is complete, open the completed segmentation file in FIJI ImageJ: “Plugins” > “ilastik” > Import HDF5.
58. Save the opened image in TIFF file format. Images following the Ilastik segmentation were shown in Figure 3. [Problem 1].
59. To convert an image to binary, the threshold must be adjusted.“Image” > “Adjust” > “Threshold” > “Auto” > “Apply” > uncheck "Calculate Threshold for each Image.
60. Repeat steps 47–59 for each channel of the original image. For multi-channel images, after individual segmentation, the channels must be merged again on FIJI using the following protocol: “Image” > “Color” > “Merge Channels”.

***Optional:*** Click on the Lookup Table option (LUT) in the Fiji toolbar to give each channel a different color for labeling.

61. Save the merged image as a TIFF file [Problem 2].

### 3D rendering-day 6

**Timing: 2 h**

This section describes how to 3-D render the binary images created from ilastik in Imaris.

62. In order to open a segmented image in Imaris, the TIFF file must be converted to an Imaris file. This can be done using the Imaris File Converter (included in the Imaris software package).
63. Make sure that the following licenses are selected in Imaris: Imaris Cell, all MeasurementPro options, and Imaris XT. The MeasurementPro option allows you to measure multichannel images on your computer.
64. In the Surpass tab, open the merged image file (currently an Imaris file).
65. In the Scene tab, create a surface (blue icon). Create the same number of surfaces as the number of channels in the image. Each surface represents a different feature. In this case, one surface represents MuSC and the other represents EC.
66. In the Surfaces tool, check the “Background Subtraction” and “Object-to-Object Statistics” check boxes; uncheck the “Classify Surfaces” check box. Do this for each surface in the multichannel image.
67. Press the green arrows to continue and a 3-D rendered surface of the feature of interest would now be created.
68. To collect data between the two surfaces, the “Statistics” tab can be clicked. Here, one can get several measurements of the surfaces (including Volume, Density, Shortest Distance to other Surfaces, and Sphericity) in the Detailed Specific Values tab. All data points collected can be saved as a .csv file and opened in Excel for further data analysis.

## Expected outcomes

**Figure 4:**
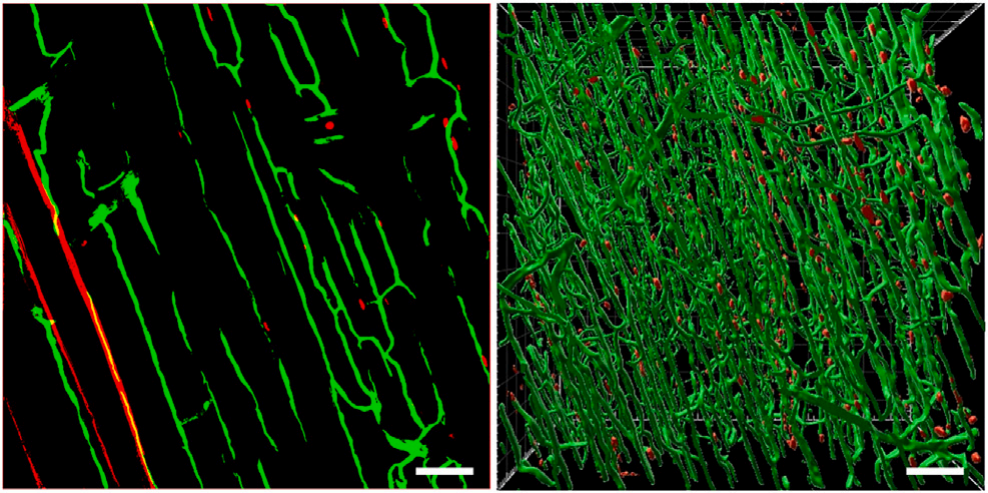
Merging of segmented channels and 3-D render on Imaris Successful merging of segmented channels (left) and 3-D render on Imaris (right) of EDL muscle. GFP(+) vascular ECs (green) and tdTomato(+) MuSCs (red). The tdTomato(+) MuSCs are closely located in the vascular ECs of the EDL muscle. Scale bars; 50 μm.

Overall, this protocol provides optimal tissue clearing, sample segmentation/3D rendering and image acquisition procedures using confocal microscopy with Ilastik and Imaris for analysis of skeletal muscle vasculature ECs and MuSCs (Figures 2, 3, and 4). This protocol can be applied to characterize various cell types within various skeletal muscle tissues of mice.

## Quantification and statistical analysis

A variety of parameters (for example, blood vessel density, nearest distance between MuSC and vasculature, MuSC sphericity, etc.) can be quantized using Imaris’ statistical capabilities. Using the .csv file that Imaris provides with object statistics, the data can be further analyzed using other data analysis software like Excel.

## Limitations

One limitation of this protocol is that the potential for there to be a high background from the tdTomato muscle fibers (especially in *mdx* mice). In *mdx* mice, the skeletal muscle cells undergo constant regeneration under normal conditions (in addition to regeneration upon stress and exercise). Therefore, when the muscle cell undergoes regeneration, the tdTomato-expressed MuSCs (muscle stem cells) undergo the differentiation/proliferation process. The result is that these cells fuse with the existing muscle fiber to repair which leads to the overall muscle fiber appearing red. This “background” makes it difficult to discern the stem cells from the muscle fibers themselves. To overcome this problem, the time between the initial TMX injection and the image acquisition can be reduced from one month to just a couple of days. In addition, background can be eliminated via careful segmentation in ilastik. If both methods are done and there is still high background, a careful filter in Imaris during the 3-D rendering can be done. MuSCs have a characteristic star/spindle shape (14). When 3-D rendering, if some objects do not display this shape, these objects can be deselected only leaving behind the actual shape of the MuSCs.

## Troubleshooting

### Problem 1

In the Image segmentation-day 6 section, the TIFF image from ilastik appears as black.

### Potential solution

If TIFF images appear black, use ImageJ’s Image > Adjust > Brightness/Contrast > Auto. Make sure the image is not a virtual stack (denoted as (V)). If it is, you will need to reopen the image as a plain TIFF file.

### Problem 2

In the Image segmentation-day 6 section, muscle fibers appear as red in addition to MuSCs after image segmentation.

### Potential solution

In Imaris, when creating the Surface tab, thresholding the larger volume muscle fibers out of range (by sliding the slider to narrow the volume range) will eliminate the presence of the larger muscle fibers.

## Resource availability

### Lead contact

Atsushi Asakura, PhD; Email: asakura@umn.edu.

### Technical contact

Atsushi Asakura, PhD; Email: asakura@umn.edu.

### Materials availability

New and unique reagents were not produced in this study.

### Data and code availability

The datasets supporting this study have not been deposited in public repositories because they are dependent on each sample. Upon request, datasets are available from the corresponding authors.

## Acknowledgments

We would like to thank the University Imaging Center in the University of Minnesota. This work was supported by Regenerative Medicine Minnesota (RMM) grants (RMM 092319 and RMM 091621) and NIH R21 grants (1R21AR078400 and 1R21AR079033).

## Author contributions

Conceptualization, M.V. and A.A.; investigation, S.K. and Y.A.; writing – original draft, S.K.; writing – review and editing, A.A.; funding acquisition, A.A.; supervision, A.A.

## Declaration of interests

The authors declare no competing interests.
